# Inhibitory effects of Myricetin derivatives on curli-dependent biofilm formation in *Escherichia coli*

**DOI:** 10.1038/s41598-018-26748-z

**Published:** 2018-05-31

**Authors:** Ken-ichi Arita-Morioka, Kunitoshi Yamanaka, Yoshimitsu Mizunoe, Yoshihiko Tanaka, Teru Ogura, Shinya Sugimoto

**Affiliations:** 1 Advanced Science Research Center, Fukuoka Dental College, Fukuoka, Japan; 2 Department of Molecular Cell Biology, Institute of Molecular Embryology and Genetics, Kumamoto University, Kumamoto, Japan; 3 Department of Bacteriology, The Jikei University School of Medicine, Tokyo, Japan; 4 Jikei Center for Biofilm Research and Technology, The Jikei University School of Medicine, Tokyo, Japan; 5 Section of Infection Biology, Department of Functional Bioscience, Fukuoka Dental College, Fukuoka, Japan

## Abstract

Biofilms are well-organised communities of microbes embedded in a self-produced extracellular matrix (e.g., curli amyloid fibers) and are associated with chronic infections. Therefore, development of anti-biofilm drugs is important to combat with these infections. Previously, we found that flavonol Myricetin inhibits curli-dependent biofilm formation by *Escherichia coli* (IC_50_ = 46.2 μM). In this study, we tested activities of seven Myricetin-derivatives to inhibit biofilm formation by *E. coli* K-12 in liquid culture. Among them, only Epigallocatechin gallate (EGCG), a major catechin in green tea, inhibited biofilm formation of K-12 (IC_50_ = 5.9 μM) more efficiently than Myricetin. Transmission electron microscopy and immunoblotting analyses demonstrated that EGCG prevented curli production by suppressing the expression of curli-related proteins. Quantitative RT-PCR analysis revealed that the transcripts of *csgA*, *csgB*, and *csgD* were significantly reduced in the presence of EGCG. Interestingly, the cellular level of RpoS, a stationary-phase specific alternative sigma factor, was reduced in the presence of EGCG, whereas the *rpoS* transcript was not affected. Antibiotic-chase experiments and genetic analyses revealed that EGCG accelerated RpoS degradation by ATP-dependent protease ClpXP in combination with its adaptor RssB. Collectively, these results provide significant insights into the development of drugs to treat chronic biofilm-associated infections.

## Introduction

Biofilms are well-organised microbial communities that attach to biotic or abiotic surfaces. Within a biofilm, microbes are embedded in a self-produced extracellular matrix composed of proteins, polysaccharides, and/or nucleic acids^1^. Since biofilm cells acquire tolerance to antimicrobial agents and host immune system, biofilms formed on tissues or implanted medical devices (e.g., catheters and orthopedic devices) can become difficult to eradicate by chemotherapeutic treatment^2^; biofilm-associated infections (e.g., catheter-related blood stream infections and prosthetic joint infections) tend to be chronic or fatal^3^. To eradicate biofilm-associated infections, effective anti-biofilm agents and novel strategies based on conceptual advances in understanding mechanisms of biofilm formation and bacterial persistence are needed.

Diverse bacterial species produce functional amyloid fibers^4,5^. Bacterial amyloids are an increasingly appreciated part of many biofilm matrices^6^. Curli are major extracellular surface amyloid fibers produced by many *Enterobacteriaceae* such as *Escherichia coli, Salmonella enterica*, and *Citrobactor spp*^7–9^. Curli fibers contribute to the initial attachment to a surface and cell-to-cell contact. Biofilms depending on curli biosynthesis are stable and their removal is difficult due to robustness of curli amyloid fibers. Importantly, curli-dependent biofilm is associated with numerous infections including urinary tract infections (UTIs), sepsis, gastroenteritis, and a complex autoimmune disease, and systemic lupus (SLE)^8,10–12^. Therefore, the development of agents against curli-dependent biofilms is an urgent and important challenge.

In *E. coli*, seven proteins encoded by two operons, curli-specific genes *BAC* (*csgBAC*) and *DEFG* (*csgDEFG*), are involved in curli expression, export, and assembly^13^. The major and minor subunits of curli are CsgA and CsgB, respectively. These proteins are synthesised in the cytoplasm, transported to the periplasm via the Sec translocon, and subsequently exported to the extracellular environment by the CsgG channel embedded in the outer membrane^14^. The exported CsgB anchors to the cell envelope and converts the unfolded state of CsgA to a β-sheet-rich amyloid polymer^15,16^. CsgC, CsgE, and CsgF play important chaperone-like functions in the transport and assembly of CsgA and CsgB^17–19^. Transcription of the *csg* operons is controlled by a complex regulatory mechanism involving CsgD and RNA polymerase sigma factor RpoS (also known as σ^38^ or σ^S^). RpoS positively regulates expression of the *csgDEFG* operon^20^ and CsgD activates transcription of the *csgBAC* operon^21^. Furthermore, transcriptional regulation of curli biogenesis is a complex process involving many other factors, which is reviewed elsewhere^22^. These regulators could be potential drug targets to combat curli-dependent biofilms.

In a previous study, we found that the molecular chaperone DnaK is absolutely required for *E. coli* biofilm formation and that a DnaK inhibitor Myricetin inhibited biofilm formation in a concentration-dependent manner (the 50% biofilm inhibition concentration: IC_50_ = 46.2 μM)^23^. In addition, Myricetin inhibited biofilm formation by Gram-positive bacterium *Staphylococcus aureus*^23,24^. On the other hand, it has been shown that Myricetin is cytotoxic towards a number of human cancer cell lines, including hepatic, skin, pancreatic and colon cancer cells^25^. Furthermore, this compound displays cytotoxicity towards normal peripheral blood mononuclear cells isolated from the blood of a healthy human^26^ and causes cellular damages to isolated guinea pig enterocytes^25,27^. Therefore, more effective and/or less cytotoxic anti-biofilm compounds should be developed.

In this report, we examined anti-biofilm activity of Myricetin-derivatives and found that Epigallocatechin gallate (EGCG) inhibited biofilm formation more efficiently than Myricetin by regulating curli biosynthesis. EGCG also effectively prevented biofilm formation by a pathogenic *E. coli* O157:H7 strain. Furthermore, EGCG promoted degradation of RpoS by ClpXP protease, which represses curli biogenesis and biofilm formation. Our findings indicate that EGCG has a potential for treatment of biofilm-related chronic infections.

## Results

### EGCG efficiently prevents *E. coli* biofilm formation

**Figure 1:**
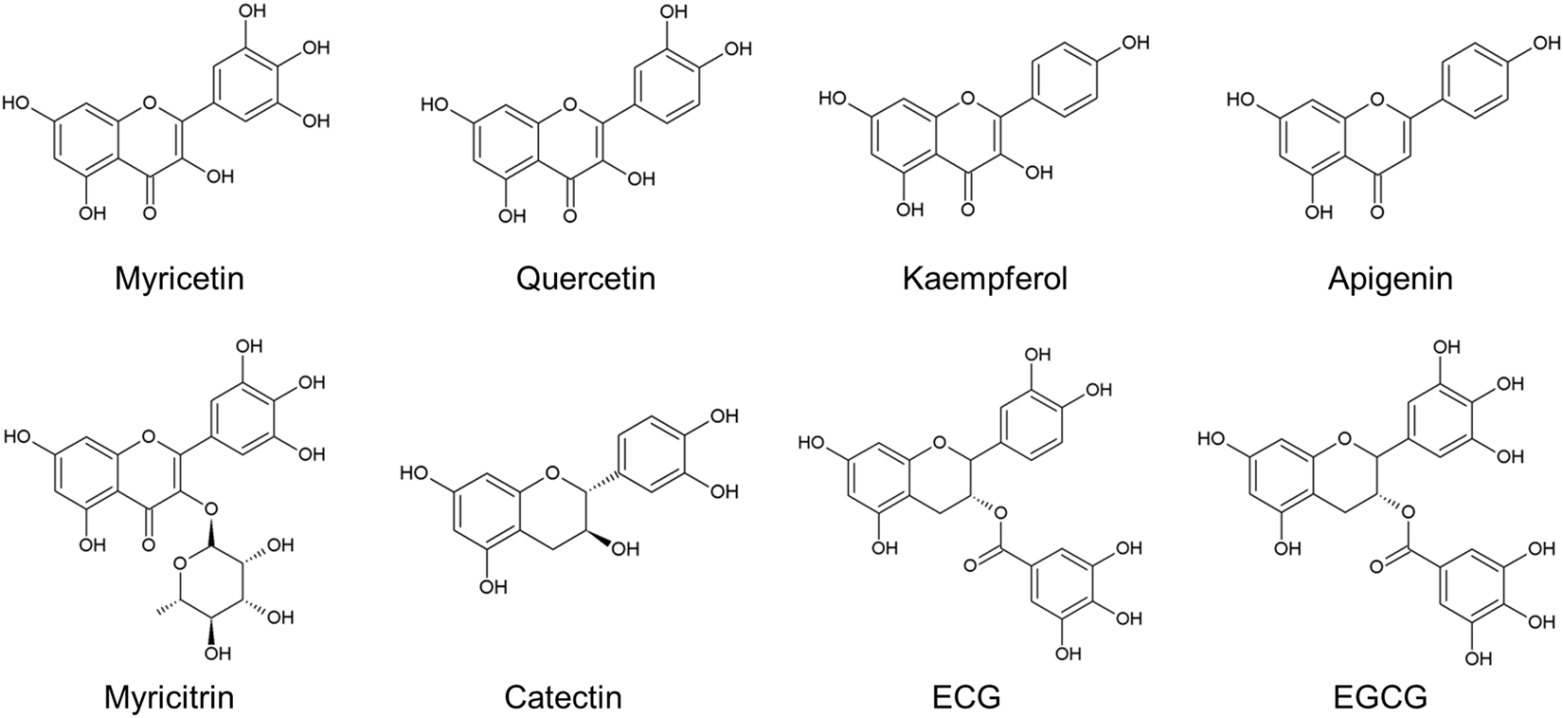
Chemical structures of Myricetin-derivatives used in this study.

**Figure 2:**
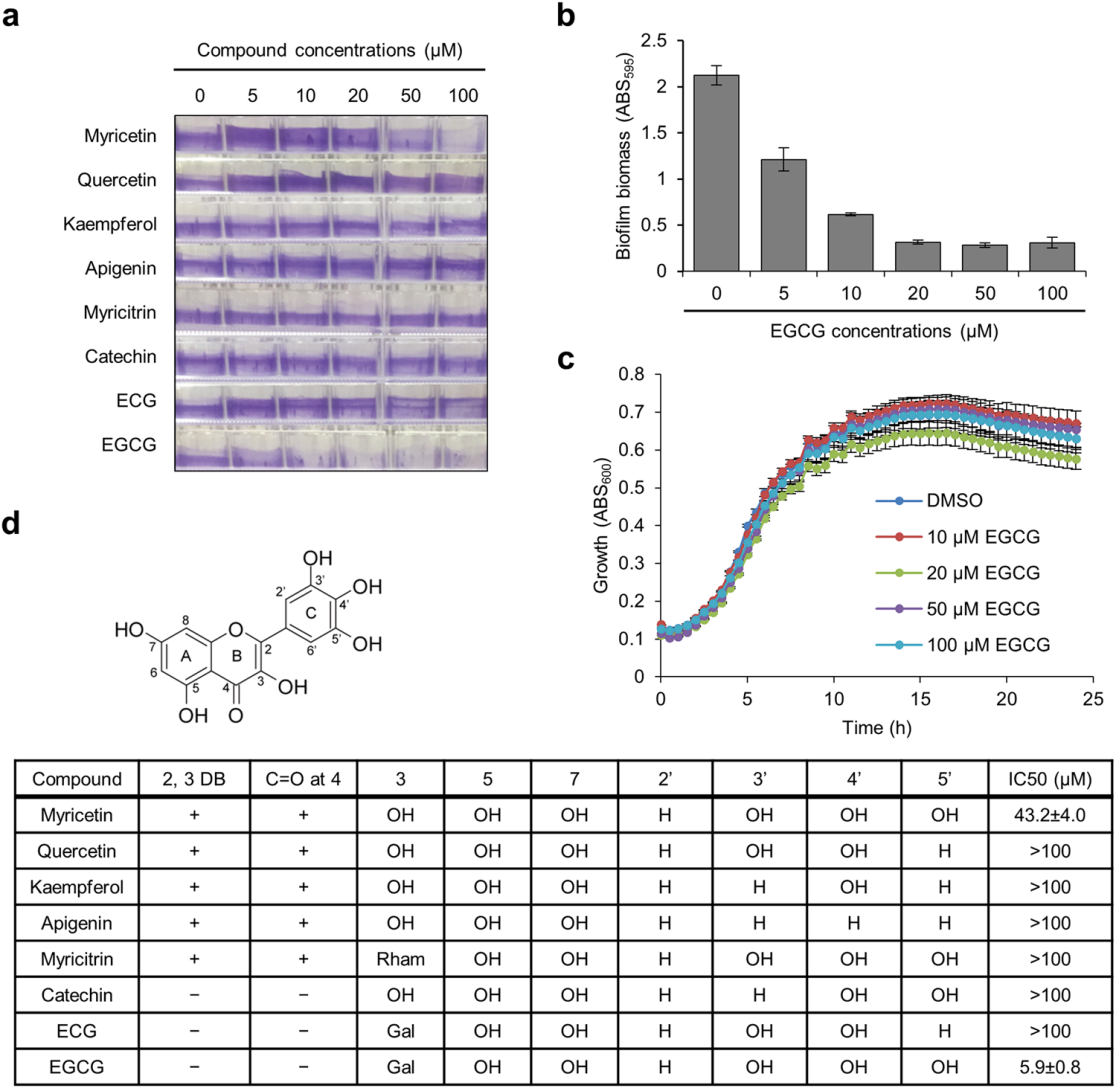
Effects of Myricetin-derivatives on *E. coli* biofilm formation. (**a**) Biofilms of *E. coli* BW25113 were formed in YESCA medium supplemented with Myricetin-derivatives at the indicated concentrations at 30 °C for 7 days. As a control, 1% DMSO without any compounds was supplemented in the medium. A photograph of the 0.2% CV-stained biofilms is shown. (**b**) Biofilm biomass was quantified by measuring absorbance at 595 nm. (**c**) Growth of *E. coli* BW25113 was monitored in the presence of EGCG at the indicated concentrations. As a control, 1% DMSO was added to the culture. (**b**,**c**) The means and standard deviations from at least triplicate determinations are represented. (**d**) Basic chemical structure of the compounds used in this study and summary of structure-function relationship among them are shown. IC_50_ was calculated as previously described^26^. DB, double bond; Rham, rhamnose; Gal, gallate.

Previously, we have demonstrated that Myricetin prevents *E. coli* biofilm formation and curli production^23^. Here, to find a more efficient inhibitor, we tested seven derivatives of Myricetin, including Apigenin, Catechin, Epicatechin gallate (ECG), EGCG, Kaempferol, Myricitrin, and Quercetin (Fig. 1). *E. coli* BW25113^28^ was cultured in YESCA medium at 30 °C for 7 days to induce aqueous biofilm formation on the side walls of 96-well polystyrene plates, in which curli-dependent biofilm can be examined^23,29,30^. Although cellulose is also one of the important biofilm matrix components in *E. coli*, some of *E. coli* K-12 strains including BW25113 have been reported not to produce cellulose due to a point mutation in BcsQ, an essential component of the *E. coli* cellulose biosynthesis apparatus that localizes at the bacterial cell pole^31–33^. Therefore, observations in this study were effects of compounds on a curli-specific phenotype. Among seven derivatives, only EGCG, which is the most abundant catechin in green tea, suppressed the biofilm formation in a concentration-dependent manner (Fig. 2a,b). In addition, EGCG showed no remarkable growth inhibition at the concentrations ranging from 10 to 100 μM (Fig. 2c). The IC_50_ of EGCG against biofilm formation by *E. coli* BW25113 was estimated to be 5.9 ± 0.8 μM (Fig. 2d), which was approximately 10-fold less than that of Myricetin (IC_50_ = 43.2 ± 4.0 μM, determined in this study).

**Figure 3:**
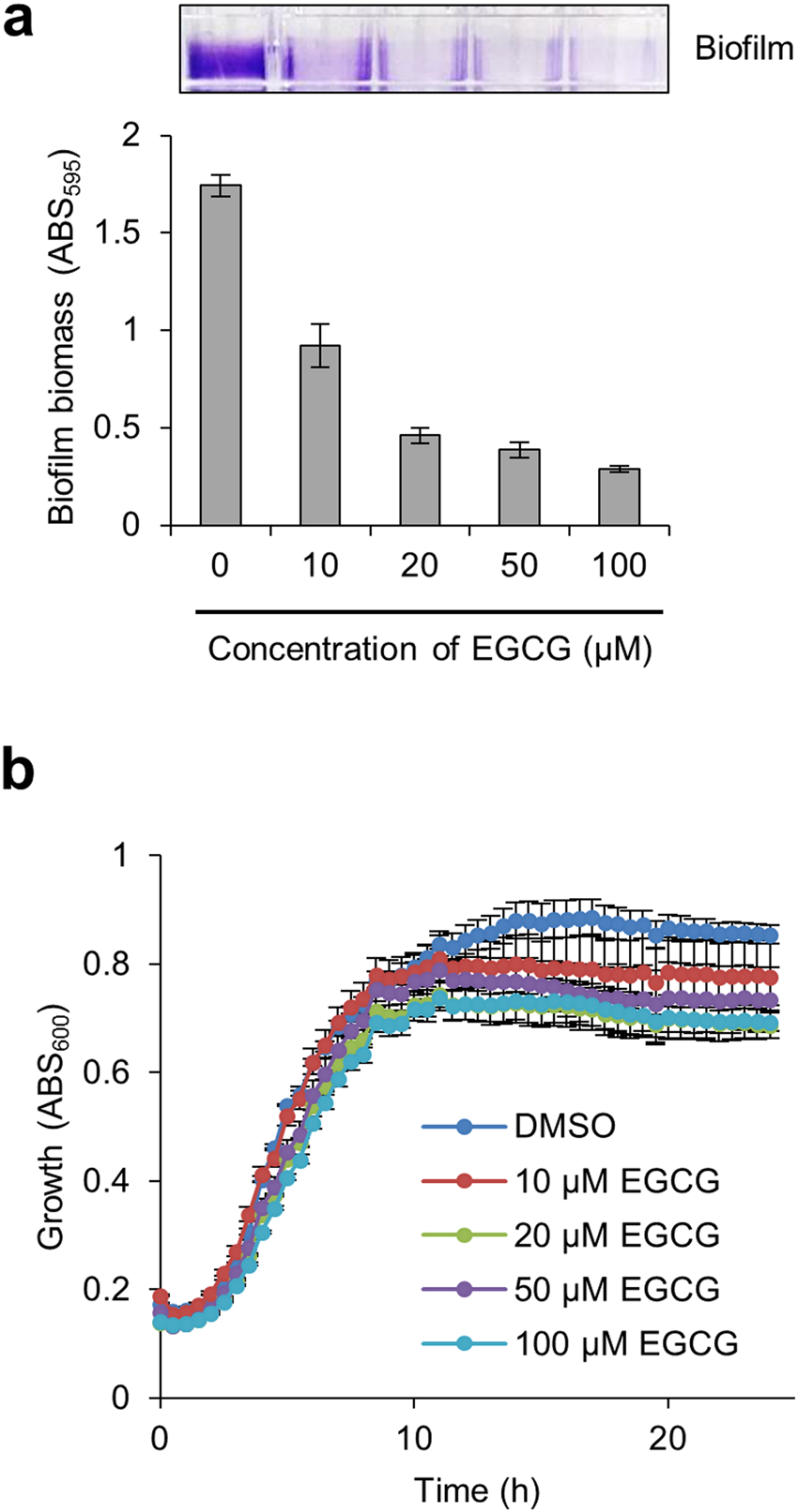
Effect of EGCG on biofilm formation by a clinically isolated strain of *E. coli*. (**a**) Biofilms of *E. coli* O157:H7 Sakai were formed in the presence of the indicated concentrations of EGCG and quantified as described in Fig. 2. (**b**) Effect of EGCG on growth of the strain was investigated at 30 °C in YESCA medium supplemented with the indicated concentrations of EGCG. As a control, 1% DMSO without any compounds was supplemented in the medium. Absorbance at 600 nm every 30 min was measured. The means and standard deviations from at least triplicate determinations are represented.

To analyse the effect of EGCG on biofilm formation by pathogenic *E. coli*, we used *E. coli* O157:H7 Sakai, a clinically isolated strain which caused a July 1996 outbreak in Japan^34^. The strain was also cultured in YESCA medium supplemented with EGCG at 30 °C for 7 days. As shown in Fig. 3a, EGCG prevented biofilm formation of Sakai in a concentration-dependent manner and the IC_50_ was 19.5 ± 5.9 μM, which was approximately three-fold higher than that against BW25113. In addition, EGCG showed no remarkable growth inhibition (Fig. 3b), as in the case of BW25113 (Fig. 2c). These results indicate that EGCG effectively prevents biofilm formation not only by an *E. coli* laboratory strain but also a pandemic strain.

### EGCG efficiently suppresses curli production in a liquid culture

**Figure 4:**
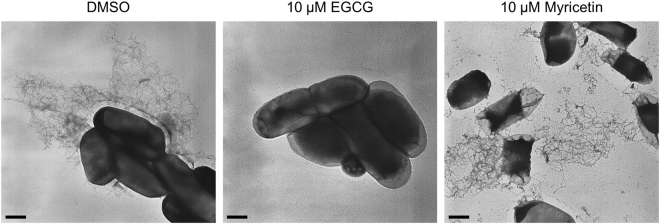
Effects of EGCG and Myricetin on curli production in liquid cultures. *E. coli* BW25113 were grown in YESCA medium in the presence of Myricetin or EGCG (10 μM each) at 30 °C for 48 h, stained with uranyl acetate, and observed by TEM. As a control, the medium was supplemented with 1% DMSO. Scales, 500 nm.

**Figure 5:**
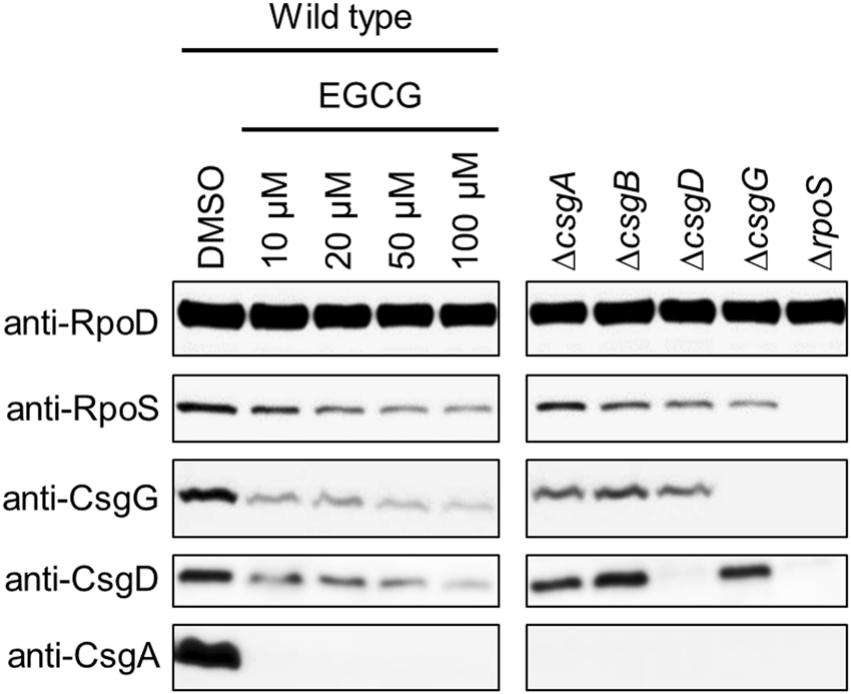
Effects of EGCG on cellular levels of curli related proteins. *E. coli* BW25113 cells were grown at 30 °C for 48 h in YESCA medium supplemented with the indicated concentrations of EGCG. As a control, the medium was supplemented with 1% DMSO. Curli-related proteins (CsgA, CsgD, CsgG, and RpoS) in the cells were analysed by immunoblotting. RpoD was detected as a loading control. Cell lysates of the indicated mutant strains grown in the presence of 1% DMSO were also used as controls. Full-size scans of immunoblots are shown in Supplementary Fig. S2.

We next analysed the curli production by transmission electron microscopy (TEM). *E. coli* BW25113 was cultured in YESCA liquid medium supplemented with EGCG or Myricetin at 30 °C for 48 h. As shown in Fig. 4, EGCG at 10 μM suppressed curli production, whereas Myricetin at the same concentration did not. Instead, much higher concentrations were required for Myricetin to suppress curli production (Supplementary Fig. S1). These results clearly indicate that EGCG is more effective for preventing curli production than Myricetin. We then examined cellular levels of curli related proteins by immunoblotting. CsgA protein was detected after depolymerized from curli fibers with hexafluoroisopropanol (HFIP). As shown in Figs 5 and S2, CsgA protein was dramatically decreased with 10 μM of EGCG, which is fully consistent with the TEM observation. EGCG also reduced the expression of CsgA in *E. coli* O157:H7 Sakai in a dose-dependent manner (Supplementary Fig. S3). Henceforth, we focused on EGCG and investigated its inhibitory mechanisms toward curli production.

To address the possibility that EGCG can suppress the CsgA polymerization on the *E. coli* cell surface, we detected unpolymerized CsgA monomers in the culture supernatant. As shown in Supplementary Fig. S4, soluble CsgA was not detected in the culture supernatant of the BW25113 wild-type strain even in the presence of EGCG, whereas it was detected in that of the Δ*csgB* strain. These results suggest that EGCG can suppress curli production at the level of transcription and/or translation rather than amyloid formation on the extracellular surface.

### Less effect of EGCG on curli production by *E. coli* grown on solid media

Next, we analysed curli production on YESCA plates supplemented with Congo Red (CR) at 30 °C for 3 days. *E. coli* BW25113 treated with EGCG at the concentrations of 10–100 μM, which were effective for preventing curli production in YESCA liquid medium, formed red colonies (Supplementary Fig. S5), suggesting that *E. coli* BW25113 produced curli on YESCA plates despite the presence of EGCG. When much higher concentrations (200–500 μM) of EGCG were supplemented, it became thinner colour in a dose-dependent manner (Supplementary Fig. S5). Immunoblotting also demonstrated that EGCG did not suppress curli production remarkably at concentrations ranging from 10 to 100 μM and that EGCG did so at much higher concentrations (200–500 μM) (Supplementary Fig. S6).

Taken together, these results indicate that EGCG inhibits curli production more efficiently than Myricetin and that the effect of EGCG is more prominent in a liquid culture rather than on a solid medium.

### EGCG suppresses the expression of curli related proteins

To further investigate the inhibitory mechanisms of EGCG toward curli production, the expression of curli-related proteins was analysed by immunoblotting. *E. coli* BW25113 cells were grown at 30 °C for 48 h in YESCA liquid medium supplemented with EGCG at the indicated concentrations. In addition to the major curli subunit CsgA, the outer membrane channel protein CsgG, and transcriptional activators CsgD and RpoS were detected. As shown in Fig. 5, the expression of CsgA, CsgG, CsgD, and RpoS were decreased in the presence of EGCG. Considering that cellular levels of CsgD and RpoS were down-regulated by EGCG (Fig. 5), the transcript levels of *csg* genes were reasonably assumed to be reduced.

**Figure 6:**
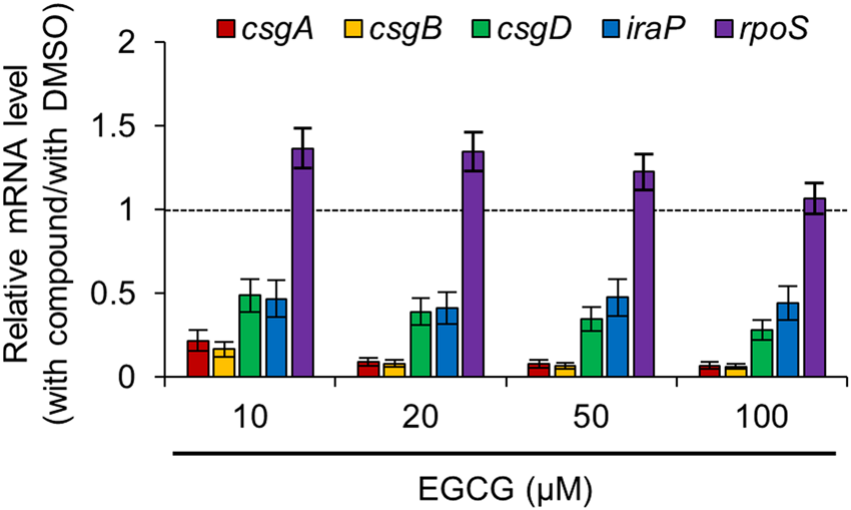
Effects of EGCG on transcription of curli-related genes. Quantitative RT-PCR analysis was performed to detect the transcripts of curli-related genes in *E. coli* BW25113 grown in YESCA liquid medium in the presence of EGCG at the indicated concentrations. As a control, the medium was supplemented with 1% DMSO. The transcript levels were normalized using *ftsZ* transcripts as internal standards. Relative mRNA levels are calculated as fold ratios relative to control cells. The means and standard deviations from at least triplicate determinations are represented.

We then measured the transcript levels of *csgA*, *csgB*, *csgD* and *rpoS* by quantitative RT-PCR. *E. coli* BW25113 cells were grown in YESCA medium supplemented with EGCG at the indicated concentrations at 30 °C for 48 h. Compared with the untreated cells, the transcript levels of *csgA*, *csgB*, and *csgD* were significantly reduced in the cells treated with EGCG (Fig. 6). On the other hand, the transcript levels of *rpoS* were not decreased with EGCG treatment (Fig. 6), suggesting that the reduced cellular levels of RpoS in the presence of EGCG were expected due to decreased stability of the protein after translation.

Collectively, these results indicate that EGCG suppresses curli-associated factors at the transcriptional and post-translational levels.

### EGCG promotes RpoS degradation

**Figure 7:**
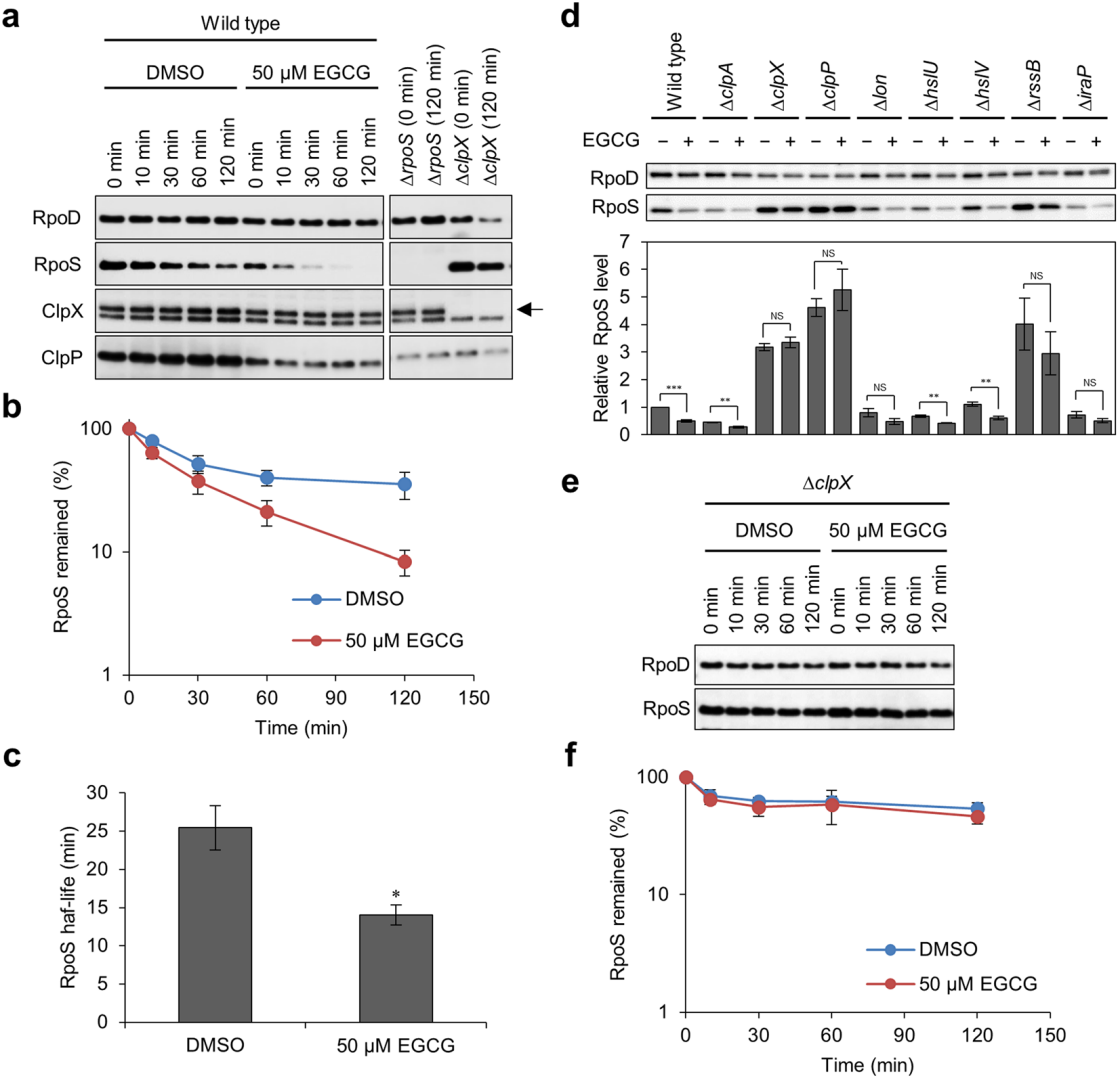
Effects of EGCG on the stability of RpoS *in vivo*. (**a**) *E. coli* BW25113 wild type and its isogenic mutant cells (∆*rpoS* and ∆*clpX*) were grown to stationary phase (24 h) in YESCA medium supplemented with 50 μM EGCG. As a control, 1% DMSO was added to the medium. At various time points after the addition of Spectinomycin, cellular proteins were analysed by SDS-PAGE and immunoblotting with anti-RpoS, anti-RpoD (loading control), anti-ClpX, and anti-ClpP antibodies. In the panels of ClpX, the upper band indicated by an arrow corresponds to ClpX and the lower one is a non-specific protein. (**b**) Band intensities of RpoS in immunoblots in **a** were measured with the LAS-4000 Image Analyser. (**c**) Half-lives of RpoS in the presence of DMSO and EGCG were calculated from data in (**b)**. The means and standard errors from at least triplicate determinations are represented. (**d**) Cellular RpoS levels in BW25113 wild type and mutant strains were analysed by immunoblotting as in **a**. These strains were harvested at 24 h without supplementation of Spectinomycin. Band intensities of RpoS in immunoblots were measured with the LAS-4000 image analyser. (**e**,**f**) Spectinomycin-chase experiments were also performed with ∆*clpX*. *P < 0.05; **P < 0.01; ***P < 0.001 (compared with DMSO control). Full-size scans of immunoblots are shown in Supplementary Figs S7–9.

To address the question of whether RpoS was destabilized in the presence of EGCG, we compared the half-life of RpoS in *E. coli* BW25113 by antibiotic chase experiments. Cells were grown to stationary phase (24 h) in the presence or absence of EGCG, Spectinomycin was added to block new protein synthesis, and at various time points the level of RpoS in each sample was determined by immunoblot analysis (Figs 7a and S7). In cells treated with 1% DMSO, the half-life of RpoS was approximately 25 min, while that in EGCG-treated cells was approximately 14 min (Fig. 7b,c). In addition, EGCG-triggered significant reduction of RpoS was observed in ∆*clpA*, ∆*hslU*, and ∆*hslV*, but neither in ∆*clpX* nor in ∆*clpP* (Figs 7d and S8). In ∆*lon*, the RpoS level was not significantly but obviously reduced by the addition of EGCG (Figs 7d and S8). Consistent with these findings, antibiotic-chase experiments revealed that EGCG did not promote RpoS degradation in ∆*clpX* (Figs 7e,f and S9). Furthermore, cellular levels of ClpX and ClpP were not increased in the presence of EGCG (Figs 7a and S10). These results indicate that EGCG can enhance the protease activity of ClpXP to degrade RpoS.

Previously, it was reported that cellular ATP levels directly control RpoS *in vivo*^35^. Therefore, we compared the ATP level between EGCG-treated and non-treated cells. As shown in Supplementary Fig. S11, there was no significant difference in the ATP level between them. This result indicated that other factor(s) can promote ClpXP activity.

Another line of experiments revealed that for RpoS degradation, ClpXP requires RssB (SprE), an adaptor protein that recognizes RpoS and delivers it to ClpXP protease^36,37^. We found that EGCG-promoted RpoS degradation was not observed in ∆*rssB* (Figs 7d and S8), indicating that degradation by RssB-ClpXP is the main pathway for RpoS degradation. It should be noted that transcript levels of *iraP*, which encodes an inhibitor of RssB^38^, were significantly reduced in the presence of EGCG (Fig. 6). In addition, although the RpoS level was reduced in ∆*iraP* compared to wild type, EGCG did not significantly accelerate RpoS degradation in ∆*iraP* (Fig. 7d and Supplementary Figs S8 and S12). Therefore, it is reasonably assumed that activation of ClpXP protease via down-regulation of IraP might be involved in the reduced cellular levels of RpoS in the presence of EGCG.

## Discussion

Previously, we have discovered that Myricetin prevents the curli-dependent biofilm formation by *E. coli*^23^. In this study, we demonstrated that the Myricetin-derivative EGCG effectively inhibited curli biosynthesis and biofilm formation by laboratory and clinically isolated *E. coli* strains (Figs 2–4). EGCG suppressed the expression of *iraP*, presumably leading to derepression of RssB-ClpXP that degrades RpoS (Figs 5–7 and Supplementary Figs S2, S7–9). Collectively, these results suggest that EGCG may be useful for treating and preventing chronic biofilm-associated infections.

The differences between EGCG and Myricetin are as follows: EGCG has (i) an extra gallate moiety appended to ring C, (ii) C–C between the positions 2 and 3 in ring C, and (iii) H at the position 4 in ring C. All or some of these alterations should contribute to the highest anti-biofilm activity of EGCG among the tested compounds. Additionally, given that ECG showed no detectable anti-biofilm activity, a hydroxyl group at the position 5′ on ring B is crucial for the activity. This is consistent with the difference between Myricetin (IC_50_ = 43.2 ± 4.0 μM) and Quercetin (IC_50_ > 100); Myricetin possesses a hydroxyl group at the positions 5′ on ring B, but Quercetin does not. On the other hand, importance of hydroxyl groups at the positions 3′ and 4′ on ring B is still unclear. Comparison between Myricetin and Myricitrin revealed that a hydroxyl group at the position 3 on ring C may be important for the activity of Myricetin as the substitution of the hydroxyl group to the rhamnose moiety abolished the activity in Myricitrin. The structural information may help to develop a more active, wide-spectrum, and less toxic agent using EGCG or Myricetin as a lead compound.

**Figure 8:**
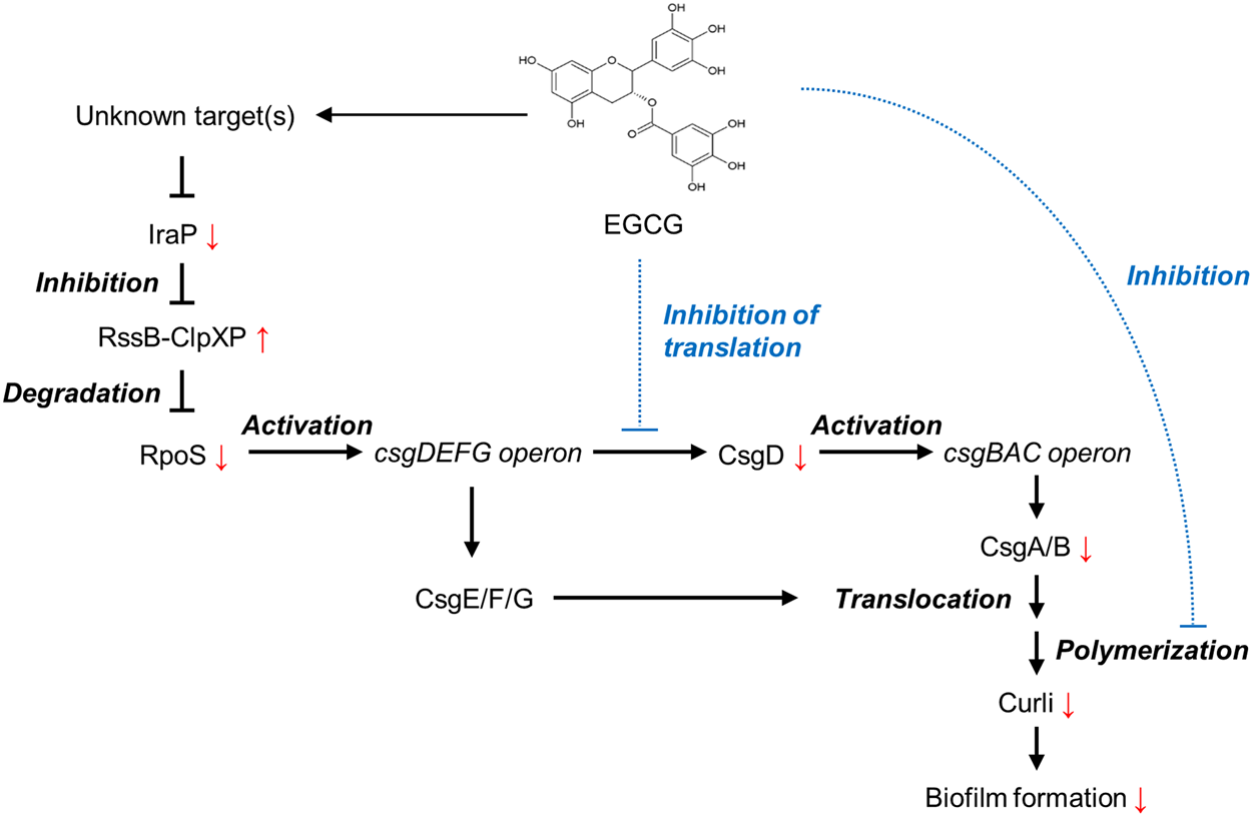
A schematic model for mode of action of EGCG. Under normal conditions, RpoS activates the *csgDEFG* operon and expressed CsgD promotes expression of the *csgBAC* operon. CsgE/F/G assists translocation of CsgA/B to outside the cell and assembly of curli amyloid fibers on the surface of the cell, leading to robust biofilm formation. EGCG may bind to unknown target molecules, which can repress the expression of *iraP*. The reduction of IraP derespresses RssB-ClpXP protease that extensively degrades RpoS, which downregulates the expression of the *csg* operons. The outcomes of the addition of EGCG are indicated as red arrows. Actions of EGCG reported by Serra *et al*.^39^ are illustrated by blue bot lines. Combination of these effects remarkably can decrease curli production and biofilm formation.

A recent report demonstrated that EGCG activates the RpoE-mediated cell envelope stress response in *E. coli*, which in turn induces the expression of the sRNA RybB^39^. Induced RybB binds to the immediate upstream region of the translation initiation codon of the *csgD* mRNA, thereby interferes with expression of CsgD^39^ (Fig. 8). Simultaneously, EGCG directly interferes with amyloid fibril formation of CsgA and CsgB^39^ (Fig. 8). It should be noted that they used higher concentrations of EGCG (12.5–400 μg/ml: 27.3–873 μM) under solid culture conditions and that higher concentrations of EGCG at more than 50 μg/ml (157 μM) were required to suppress curli production effectively. However, effects of EGCG on biofilm formation in liquid cultures were not examined. In the present study, we found that much lower concentrations of EGCG (<10 μM) were sufficient for preventing curli production and biofilm formation under liquid culture conditions (Figs 2–5). We also confirmed that lower concentrations of EGCG (1–100 μM) were insufficient for inhibiting curli production on YESCA plates (Supplementary Figs S5 and S6). Much weaker effects of EGCG under solid culture conditions were probably due to the inefficient transfer of EGCG from the bottom to the top of macrocolonies, where the abundant production of curli was observed^40^. In contrast, bacterial cells were surrounded by EGCG in the liquid culture, and thus, EGCG may efficiently access to target molecules on the surface or inside cells. In the recent paper^39^, the cellular level of RpoS was only slightly affected by EGCG under solid culture conditions. In contrast, remarkable reduction of RpoS levels (~65%) was observed even when *E. coli* cells were grown on agar plates containing 200–500 μM EGCG (Supplementary Fig. S6). The discrepancy between the previous study^39^ and the present study might be due to differences in *E. coli* strains (BW25113 vs W3110) and culture conditions (YESCA at 30 °C vs salt-free LB at 28 °C).

Our results indicated that EGCG reduced the cellular levels of CsgD as well as RpoS (Figs 5 and S2), both of which play a crucial regulatory role in the expression of the *csg* operons. Therefore, curli production was inhibited by EGCG at the transcriptional level (Fig. 6) rather than post-translational levels (e.g., polymerization of CsgA into amyloid fibers as shown in Supplementary Fig. S4) under the conditions in this study. It is important to mention that our present results showed significant decrease in the level of RpoS by EGCG under the conditions tested (Figs 5 and S2), although the transcript level of *rpoS* was not affected (Fig. 6). Antibiotic-chase experiments revealed that EGCG accelerated RpoS degradation by RssB-ClpXP (Figs 7 and S7–9). Interestingly, transcript levels of *iraP* encoding the inhibitor for RssB-ClpXP protease were significantly reduced in the presence of EGCG (Fig. 6), which may contribute to the activation of the RssB-ClpXP proteolytic pathway (Fig. 8). Therefore, it seems likely that EGCG reduced the cellular level of RpoS at the post-translational level. It is important to mention that RpoS is also regulated at a post-transcriptional level. Small RNAs (DsrA, RprA, and ArcZ) are known to promote translation of *rpoS* mRNA^22^. In addition, it has recently been reported that these small RNAs prevent Rho-dependent premature transcription termination of *rpoS*, leading to the increase in the production of mature *rpoS* mRNA^41^. Therefore, these small RNAs participate in the expression of RpoS not only at the post-transcriptional level but also at the transcriptional level. Our data showed that the levels of *rpoS* mRNA were not reduced by the addition of EGCG, suggesting that EGCG may not reduce the levels of these small RNAs. Nevertheless, EGCG can affect curli production at multiple stages such as the level of RpoS, the translation of CsgD via the sRNA RybB^39^ and other unknown pathway(s). Although a current model for mode of action of EGCG is schematically illustrated in Fig. 8, precise molecular mechanisms underlying how EGCG suppresses curli production are still largely unknown. Identification of direct target(s) of EGCG will provide significant insight into its mode of action and might be helpful for development of drugs to prevent and cure biofilm-associated infections.

Previously, it was shown that EGCG has antimicrobial effects against a variety of bacteria via a variety of antimicrobial mechanisms (e.g., damage to the bacterial cell membrane, inhibition of fatty acid synthesis, and inhibition of enzyme activity)^42^. EGCG was also shown to inhibit biofilm formation by various bacterial species including *Staphylococcus aureus*^43^, *Staphylococcus epidermidis*^43^, *Streptococcus mutans*^44^, *Porphyromonas gingivalis*^45^, *Pseudomonas aeruginosa*^46^, and *Fusobacterium nucleatum*^47^ at sub-minimal inhibitory concentrations for cell growth. Some of these bacteria produce extracellular amyloid fibers like curli^4,5^, suggesting that one of the targets of EGCG is amyloid formation as in the case that fiber formation of pathogenic amyloids involved in protein misfolding disorders, including Alzheimer’s and Parkinson’s diseases is inhibited by EGCG^48,49^. In addition, EGCG suppresses several virulence factors (toxins and enzymes) produced by pathogenic bacteria^44,47,50^. Taken altogether, EGCG has multiple targets for inhibiting biofilm formation and can be applicable to diverse purposes that are not limited to against biofilm-associated infections.

## Methods

### Bacterial strains

Wild-type *E. coli* K-12 BW25113 and its isogenic mutants Δ*csgA*, Δ*csgB*, Δ*csgD*, Δ*csgG*, Δ*rpoS*, Δ*clpA*, Δ*clpX*, Δ*clpP*, Δ*lon*, Δ*hslU*, Δ*hslV*, Δ*rssB*, and Δ*iraP* (Keio collection)^28^ were provided by National BioResource Project (NBRP) in Japan. *E. coli* O157:H7 Sakai^34^ was also used in this study to examine activity of EGCG to prevent biofilm formation by a pathogenic *E. coli* strain.

### Flavonoids

Apigenin, Catechin, ECG, EGCG, Kaempferol, Myricetin, and Myricitrin were purchased from Tokyo Kasei (Tokyo, Japan), and Quercetin was from Wako Pure Chemical Industries (Osaka, Japan). Stock solutions of these compounds were prepared in 100% dimethyl sulfoxide (DMSO) at 100-times higher concentrations than the indicated final concentrations and diluted 100-fold in culture media to yield the indicated concentrations. Therefore, tested media contained 1% DMSO.

### Biofilm formation

Biofilm formation was analysed at 30 °C for 7 days in YESCA medium (1% casamino acid and 0.1% yeast extract) supplemented with the indicated concentrations of flavonoids in 96-well polystyrene plates as previously described^23^. Briefly, culture medium and planktonic cells were removed, and the biofilms bound to the surface of the wells were washed with phosphate-buffered saline (PBS). The residual biofilms were stained with 0.2% crystal violet, rinsed twice with PBS. After taking photographs of biofilms, crystal violet was extracted from biofilms with 70% ethanol. Absorbance at 595 nm of the extracted solutions was measured by using a spectrophotometer (Wallac 1420 ARVO MX; PerkinElmer, Boston, MA, USA).

### Growth assay

*E. coli* strains were grown in LB medium overnight at 30 °C. The cultures were diluted 1,000-fold in YESCA medium supplemented with the indicated concentrations of EGCG or Myricetin, and 1% DMSO, and 200-μl aliquots were cultured in 96-well flat-bottomed polystyrene plates (Corning, Corning, NY, USA) at 30 °C for 24 h with shaking. The absorbance of each culture at 600 nm was measured every 30 min for 24 h using a Bio Microplate Reader HiTS (Scinics Corp., Tokyo, Japan).

### CR-binding assay

*E. coli* strains were grown in LB medium overnight at 30 °C with shaking. Five microliters of the overnight cultures were spotted on YESCA plates containing 2% agar, 10 μg/ml CR, 10 μg/ml Coomassie Brilliant Blue G-250, the indicated concentrations of EGCG, and 1% DMSO. Plates were incubated at 30 °C for 3 days.

### TEM

Curli produced in the extracellular matrix of *E. coli* was visualized by TEM. *E. coli* strains were grown at 30 °C in YESCA medium supplemented with EGCG or Myricetin at the indicated concentrations. After 48-h incubation, samples were stained with 2% uranyl acetate and observed with a transmission electron microscope (JEM-1400; JEOL, Tokyo, Japan) at a voltage of 80 kV.

### RT-PCR

*E. coli* BW25113 cells were grown in LB medium overnight at 30 °C with shaking. Aliquots (5 μl) of the cultures were diluted into 5 ml fresh YESCA medium supplemented with EGCG at the indicated concentrations in 6-well plates and incubated at 30 °C for 48 h. Total RNA was purified using the QIAGEN RNeasy Mini Kit (Qiagen, Hilden, Germany) according to the manufacture’s instruction. cDNA was generated using the Prime Script II 1st strand cDNA Synthesis Kit (Takara, Otsu, Japan) according to the manufacture’s instruction. The transcript levels of *csgA, csgB, csgD*, *rpoS*, and *iraP* were measured by RT-PCR using primer sets RT-csgA-F/RT-csgA-R, RT-csgB-F/RT-csgB-R, RT-csgD-F/RT-csgD-R, RT-rpoS-F/RT-rpoS-R, and RT-iraP-F/RT-iraP-R, respectively (Table S1). As an internal control, the *ftsZ* transcript level was also measured using primers RT-ftsZ-F and RT-ftsZ-R (Table S1). RT-PCR reactions were performed on a CFX96 Real-Time System (Bio-Rad Laboratories, Hercules, CA, USA).

### Antibodies

Antibodies used in this study were prepared as recently described^51^. Briefly, rabbit anti-CsgA, and -CsgG antisera were developed and purified using antigen-conjugated affinity resins by Medical Biological Laboratories Co. (Aichi, Japan). Rabbit anti-CsgD-Myc-His was developed and purified with CsgD-Myc-His conjugated affinity resin by Eurofins Genomics. Rabbit anti-ClpX and anti-ClpP antisera were developed by Eurofins Genomics. Mouse monoclonal anti-RpoS and -RpoD antibodies were purchased from Abcam (Cambridge, MA, USA). Horseradish peroxidase (HRP)-conjugated goat anti-rabbit and anti-mouse IgG (Bio-Rad Laboratories) antibodies were used as secondary antibodies.

### Antibiotic-chase experiments

*E. coli* BW25113 wild type and its isogenic mutant cells were grown at 30 °C for 24 h in 5 ml YESCA medium supplemented with 50 μl of 5 mM EGCG dissolved in 100% DMSO (final concentrations: 50 μM EGCG and 1% DMSO). As a control, only 100% DMSO was added to the final concentration of 1%. At various times after addition of 200 μg/ml Spectinomycin, the samples were mixed with the same volume of 2× sodium dodecyl sulfate (SDS) sample buffer [150 mM Tris-HCl (pH 6.8), 4% SDS, 20% glycerol, and 10% 2-mercaptoethanol] and immediately frozen on dry ice or at −80 °C. After boiling at 95 °C for 5 min, cellular proteins were analysed by immunoblotting with anti-RpoS, anti-RpoD (loading control), anti-ClpX, and anti-ClpP antibodies as described below.

### Immunoblotting

To detect CsgA monomers produced in YESCA liquid medium, curli fibers were depolymerized into subunits by treatment with HFIP prior to SDS-polyacrylamide gel electrophoresis (PAGE). Bacterial cells grown in 20 ml YESCA medium including wall-attached cells were harvested by centrifugation at 8,000 × *g* for 30 min at 4 °C and resuspended in 400 μl STE buffer [10 mM Tris-HCl (pH 8.0), 100 mM NaCl, and 2 mM EDTA] and aliquots (10 μl) were mixed with 100 μl HFIP. After sonication in a water bath for 10 min at room temperature, samples were vacuum dried with a SpeedVac vacuum concentrator (Thermo Fisher Scientific, Waltham, MA, USA) at 45 °C for more than 30 min. Dried samples were dissolved in 20 μl of 8 M urea solution and sonicated in a water bath for 5 min at room temperature. Solutions were mixed with the equal volume of 2 × SDS sample buffer and aliquots (10 μl) were separated by SDS- PAGE on SDS-15% polyacrylamide gels.

To detect CsgA monomers in culture supernatants, the biofilm culture supernatants (2 ml) prepared as described above were mixed with 100% ice-cold acetone (5 ml), incubated overnight at −30 °C, and centrifuged at 10,000 × *g* for 30 min at 4 °C. Pellets were washed with 100% ice-cold acetone (1 ml), centrifuged at 10,000 × *g* for 10 min at 4 °C, and mixed with 200 μl HFIP to dissolve aggregates formed during sample preparation. After sonication in a water bath for 10 min at room temperature, samples were vacuum dried with a SpeedVac vacuum concentrator at 45 °C for 30 min. Dried samples were dissolved in 40 μl of 8 M urea solution and mixed by vortex. Solutions were mixed with the equal volume of 2 × SDS sample buffer and aliquots (10 μl) were separated by SDS-PAGE on SDS-15% polyacrylamide gels.

To detect CsgA monomers produced on YESCA agar plates, curli fibers were depolymerized as described elsewhere^51^. Briefly, *E. coli* cells were harvested from colonies grown on YESCA plates at 30 °C for 3 days using a scraper and resuspended in STE buffer (10 μl per 1 mg cells) and mixed with 100 μl HFIP. After brief sonication, samples were vacuum dried with a SpeedVac vacuum concentrator and dried samples were dissolved in 20 μl of 8 M urea solution. Solutions were mixed with the equal volume of 2 × SDS sample buffer and aliquots (5 μl) were separated by SDS-PAGE on SDS-15% polyacrylamide gels except for heat treatment of samples.

After SDS-PAGE, proteins were transferred to polyvinylidene difluoride membranes using the iBlot 2 dry blotting system (Thermo Fisher Scientific) according to the manufacturer’s instructions. Membranes were treated with blocking solution composed of 1–5% skimmed milk in Tris-buffered saline containing 0.1% (v/v) Tween 20 (TBS-T) for at least 1 h or overnight at 25 °C. After gentle washing with TBS-T, the membrane was probed with antibodies against CsgA (1/200), CsgD (1/2,000), CsgG (1/1,000), RpoD (1/4,000), RpoS (1/2,000), ClpX (1/10,000), or ClpP (1/10,000) diluted in CanGet Signal 1 (Toyobo) for at least 1 h or overnight at 25 °C. Membranes were washed twice with TBS-T. To detect CsgA, CsgD, CsgG, ClpX, and ClpP, membranes were then incubated with HRP-conjugated goat anti-rabbit IgG antibody (1/50,000 in CanGet Signal 2; Toyobo) for 1 h at 25 °C. To detect RpoS and RpoD, membranes were incubated with HRP-conjugated goat anti-mouse IgG antibody (1/2,000 in CanGet Signal 2) for 1 h at 25 °C. After three washes with TBS-T, signals were detected using the ECL Prime Western Blotting Detection Reagent (GE Healthcare, Pittsburgh, PA, USA) and LAS-4000 Image Analyser (GE Healthcare).

### Quantification of cellular ATP levels

*E. coli* BW25113 cells were grown at 30 °C for 24 h in 5 ml YESCA medium supplemented with 50 μM EGCG. As a control, 1% DMSO was added to the medium. For quantification of ATP, bacterial cultures were mixed with the equal volume of the BacTiter-Glo Microbial Cell Viability Assay kit (Promega). After incubation at 25 °C for 5 min with shaking, luminescence was recorded on a microtiter plate reader (Infinite F200 Pro, Tecan, Männedorf, Switzerland).

### Statistical analysis

Student’s t test was used to assess half-life and cellular level of RpoS using Microsoft Excel software. For all analyses, a *P* value of <0.05 was considered statistically significant.

## Electronic supplementary material

Supplementary Information
