# Supplementary material for: Inhibitory effects of Myricetin derivatives on curli-dependent biofilm formation in Escherichia coli
Source: Sci Rep. 2018 May 31;8:8452. doi: 10.1038/s41598-018-26748-z (PMC5981455; doi:10.1038/s41598-018-26748-z)
Supplement: Supplementary file 1 — Supplementary Information [file 41598_2018_26748_MOESM1_ESM.pdf]

Supplementary Information

Inhibitory effects of Myricetin derivatives on curli-dependent biofilm formation in *Escherichia coli*

Ken-ichi Arita-Morioka[1,2,†], Kunitoshi Yamanaka[2], Yoshimitsu Mizunoe[3,4], Yoshihiko Tanaka[1,5], Teru Ogura[2] , Shinya Sugimoto[3,4,†,*]

[1]Advanced Science Research Center, Fukuoka Dental College, Fukuoka, Japan.

[2]Department of Molecular Cell Biology, Institute of Molecular Embryology and Genetics, Kumamoto University, Kumamoto, Japan.

[3]Department of Bacteriology, The Jikei University School of Medicine, Tokyo, Japan.

[4]Jikei Center for Biofilm Research and Technology, The Jikei University School of Medicine, Tokyo, Japan.

[5]Section of Infection Biology, Department of Functional Bioscience, Fukuoka Dental College, Fukuoka, Japan.

[†]These authors contributed equally to the work

*Correspondence should be addressed to S.S. (email: ssugimoto@jikei.ac.jp)

Supplementary Table S1. Oligonucleotide primers used in this study.

**Primer name**	**Sequence (5' to 3')**
RT-csgA-F	TCTGGCAGGTGTTGTTCCTC
RT-csgA-R	CCACCACCATGCTGGGTAAT
RT-csgB-F	TAACAATACTGGGTGCGCCT
RT-csgB-R	GGTCAATCTTTGCCCGGTTG
RT-csgD-F	TCTCGTTATTAGACGCGCCG
RT-csgD-R	CAATGGATTGCAAGGCGTCC
RT-rpoS-F	GGTAAAAATTGCCCGCCGTT
RT-rpoS-R	AATCGCCCGTTCAATCGTCT
RT-iraP-F	ACTGTGTGCGCAGGTAGAAG
RT-iraP-R	CTTTACGTAATCGCGCAGCA
RT-ftsZ-F	ATGGAACTTACCAATGACGCG
RT-ftsZ-R	TCAACACCTTCAATGCGCTC

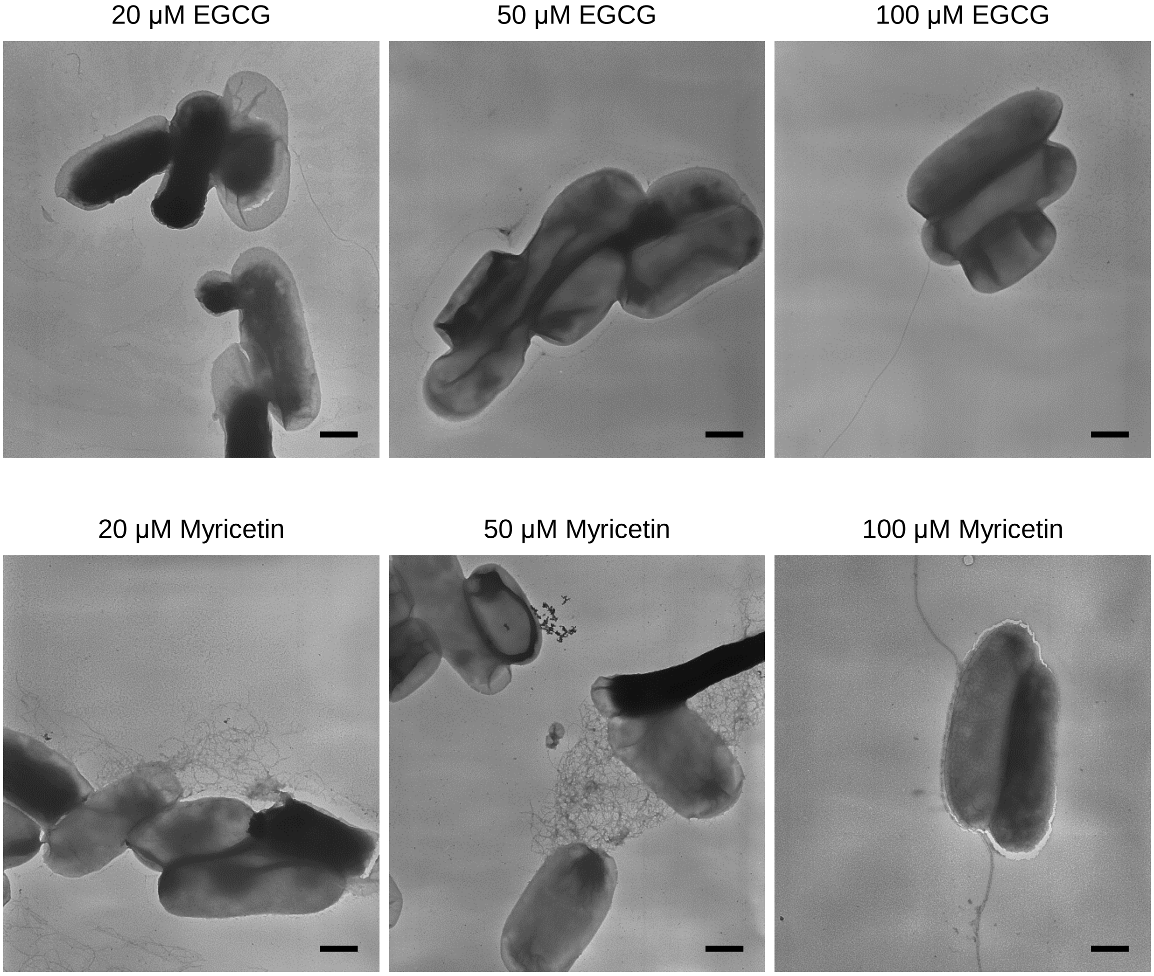

Supplementary Figure S1. Effects of EGCG and Myricetin on curli production. *E. coli* BW25113 grown at 30°C for 48 h in YESCA medium in the presence of Myricetin or EGCG at the indicated concentrations were observed by TEM as described in Figure 4. Scales, 500 nm.

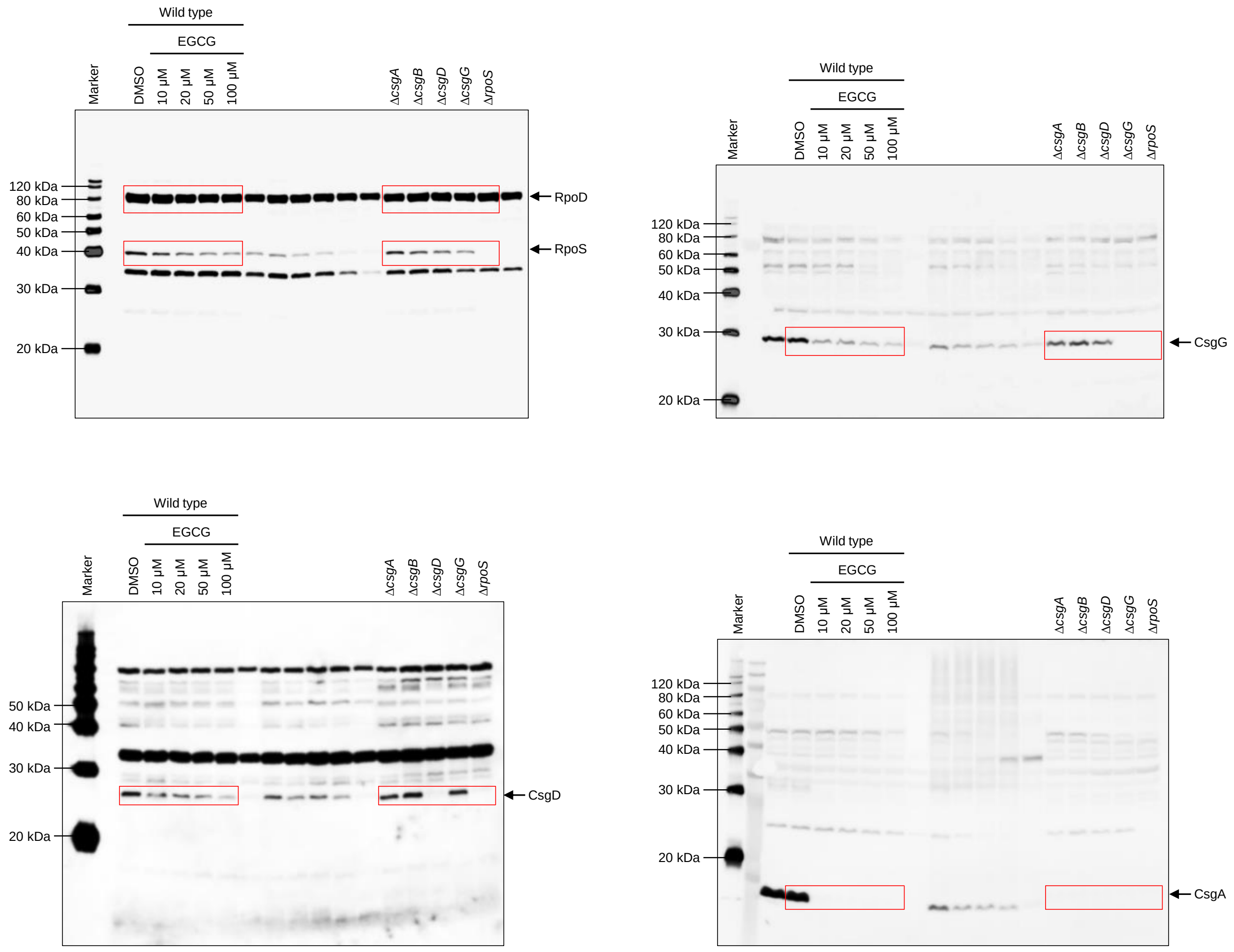

Supplementary Figure S2. Full blots for detection of RpoD, RpoS, CsgG, CsgD and CsgA. Portions (red squares) are used in Figure 5. The positions of molecular size markers are shown to the left of the blots.

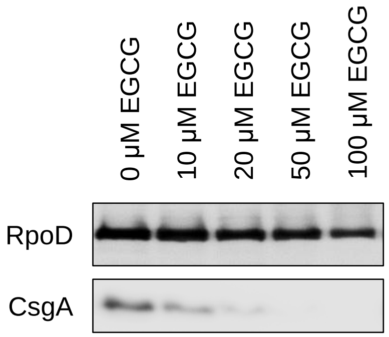

Supplementary Figure S3. Detection of CsgA produced by the pathogenic *E. coli* clinical strain grown in liquid medium. CsgA was detected by immunoblotting in *E. coli* O157:H7 Sakai grown at 30°C for 48 h in YESCA medium supplemented with EGCG at the indicated concentrations. To depolymerize curli into subunits, samples were treated with HFIP before SDS-PAGE. As a control, the medium was supplemented with 1% DMSO. RpoD was detected as a loading control.

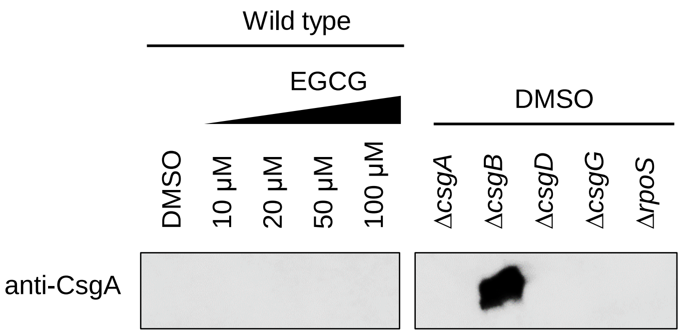

Supplementary Figure S4. Detection of CsgA in culture supernatants. Culture supernatants under biofilm forming conditions in the presence of EGCG at the indicated concentrations were used for detecting CsgA by immunoblotting. As controls, YESCA medium supplemented with 1% DMSO and the indicated BW25113 isogenic mutants were used. The Δ*csgB* strain was used as a positive control since it releases CsgA extracellularly.

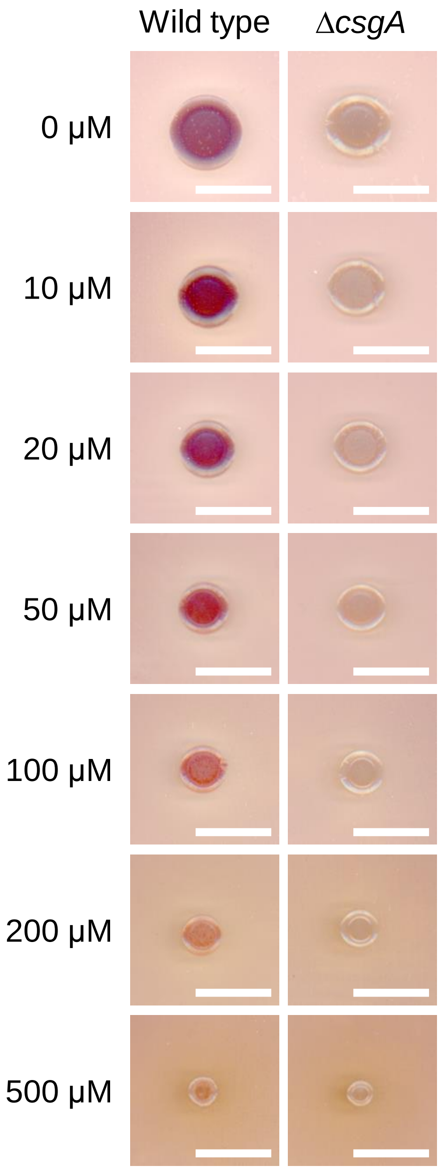

Supplementary Figure S5. Curli production on CR agar plates. Overnight culture of wild-type *E. coli* BW25113 were spotted on CR agar plates without supplementation (1% DMSO) or supplemented with EGCG at the indicated concentrations. Plates were incubated at 30°C for 3 days. As a negative control, the isogenic Δ*csg*A strain was used. Scales, 1 cm.

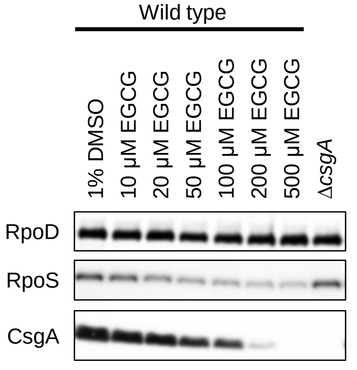

Supplementary Figure S6. Detection of CsgA produced by *E. coli* cells grown on solid medium. CsgA was detected by immunoblotting in *E. coli* BW25113 grown at 30°C for 3 days on YESCA plates supplemented with EGCG at the indicated concentrations. To depolymerize curli into subunits, samples were treated with HFIP before SDS-PAGE. As a control, plates were supplemented with 1% DMSO. RpoD was detected as a loading control.

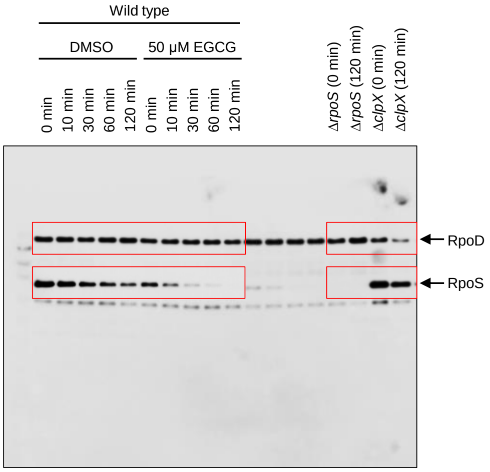

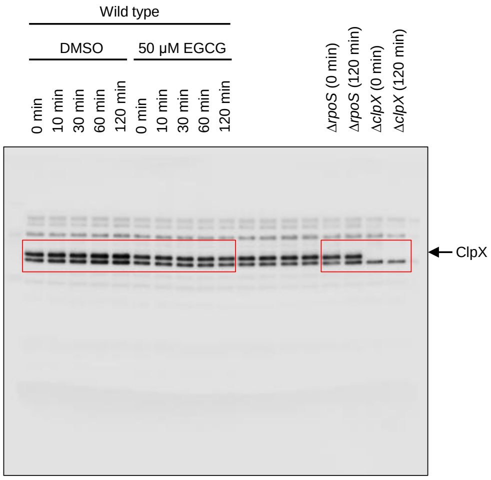

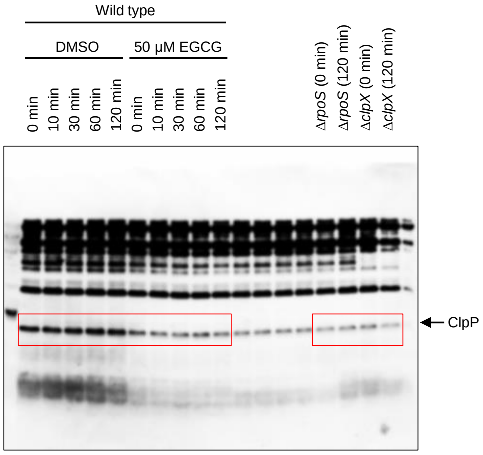

Supplementary Figure S7. Full blots for detection of RpoD, RpoS, CsgG, CsgD and CsgA. Portions (red squares) are used in Figure 7a.

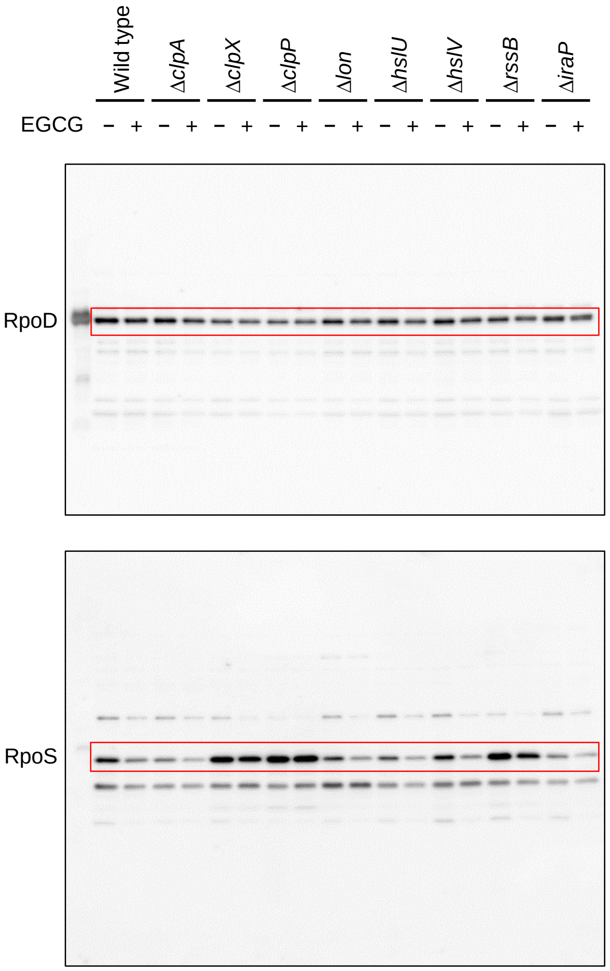

Supplementary Figure S8. Full blots for detection of RpoD, RpoS, CsgG, CsgD and CsgA. Portions (red squares) are used in Figure 7d.

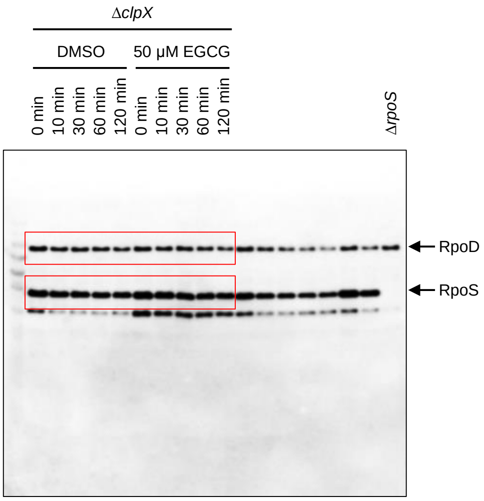

Supplementary Figure S9. Full blots for detection of RpoD, RpoS, CsgG, CsgD and CsgA. Portions (red squares) are used in Figure 7e.

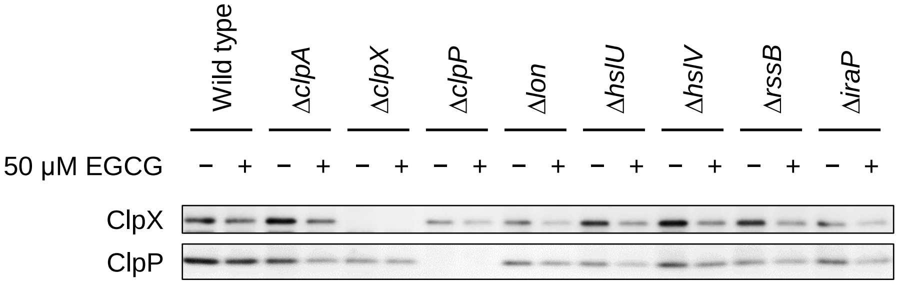

Supplementary Figure S10. Effect of EGCG on cellular levels of ClpXP. *E. coli* BW25113 wild type and the indicated isogenic mutants were grown to stationary phase (24 h) in YESCA medium supplemented (+) or non-supplemented (−) with 50 µM EGCG. Cellular proteins were analyzed by SDS-PAGE and immunoblotting with anti-ClpX and anti-ClpP as in Figure 7.

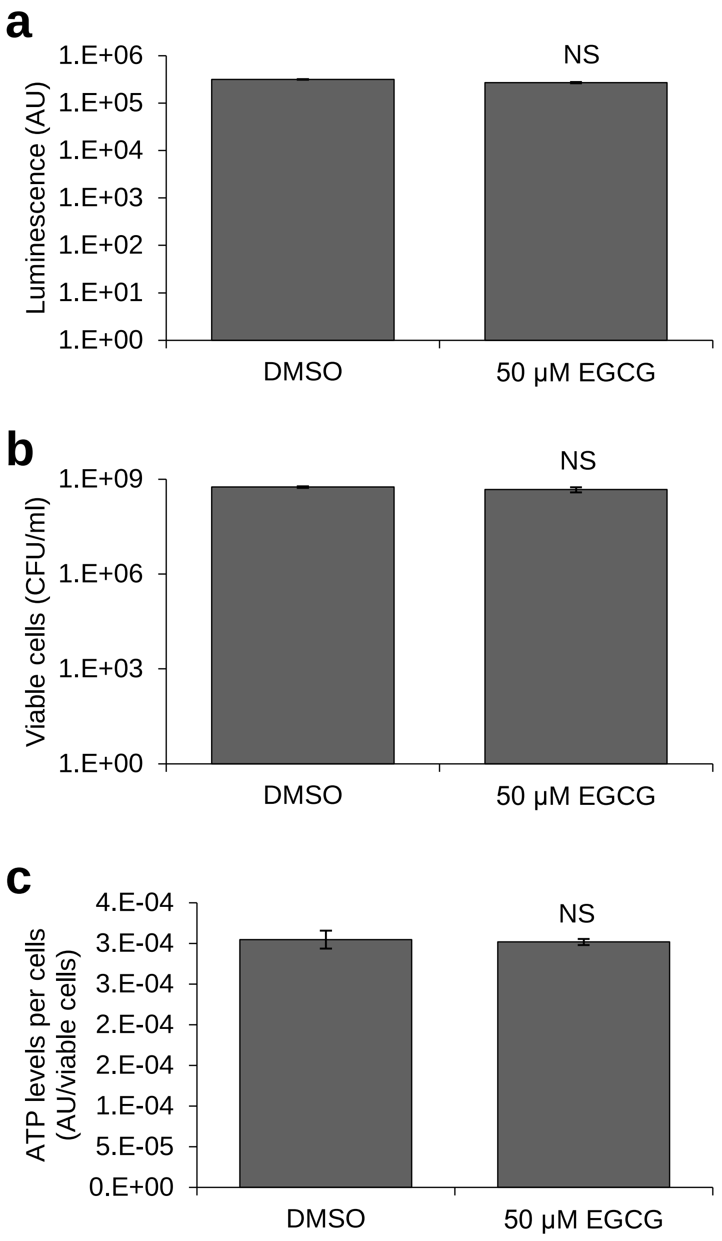

Supplementary Figure S11. Effect of EGCG on intracellular ATP concentration. (**a**) *E. coli* BW25113 cells were grown in YESCA medium supplemented with 50 μM EGCG. As a control, 1% DMSO was supplemented into the medium. After cultivation at 30°C for 24 h, cellular ATP levels were quantified using the BacTiter-Glo Microbial Cell Viability Assay kit (Promega). (**b**) Viable cells were quantified by measuring colony forming unit per 1 ml culture (CFU/ml) after cultivation at 30°C for 24 h in YESCA medium supplemented with 1% DMSO or 50 μM EGCG. (**c**) The intracellular ATP levels were calculated by dividing luminescence (AU) in **a** with CFU/ml in **b**. The means and standard deviations from triplicate determinations are represented. NS, not significant.

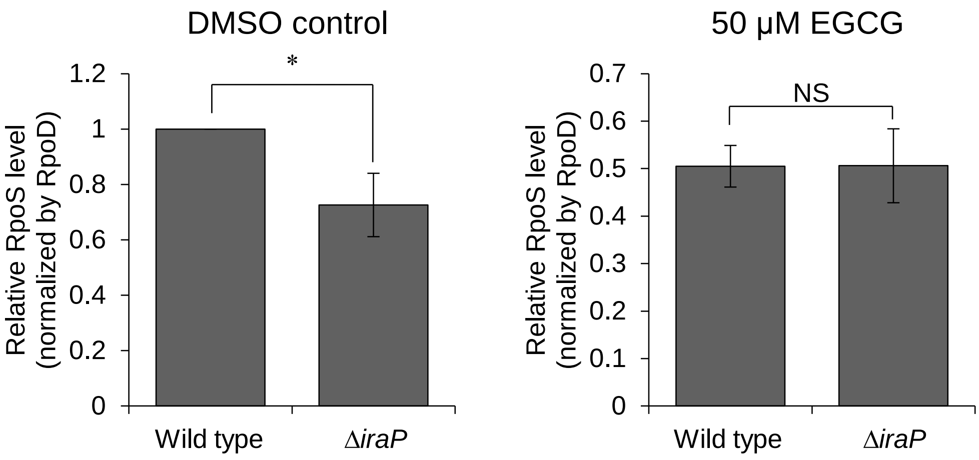

Supplementary Figure S12. Quantification of cellular RpoS levels. Band intensities of RpoS in wild type and Δ*iraP* in the absence (DMSO control) and presence of EGCG were quantified as described in Fig. 7d.
